# Low-count whole-body PET with deep learning in a multicenter and externally validated study

**DOI:** 10.1038/s41746-021-00497-2

**Published:** 2021-08-23

**Authors:** Akshay S. Chaudhari, Erik Mittra, Guido A. Davidzon, Praveen Gulaka, Harsh Gandhi, Adam Brown, Tao Zhang, Shyam Srinivas, Enhao Gong, Greg Zaharchuk, Hossein Jadvar

**Affiliations:** 1 Department of Radiology, Stanford University, Palo Alto, CA USA; 2 Department of Biomedical Data Science, Stanford University, Stanford, CA USA; 3 Subtle Medical, Menlo Park, CA USA; 4 Division of Diagnostic Radiology, Oregon Health & Science University, Portland, OR USA; 5 Department of Radiology, University of Pittsburgh Medical Center, Pittsburgh, PA USA; 6 Department of Radiology, University of Southern California, Los Angeles, CA USA

**Keywords:** Cancer imaging, Radionuclide imaging, Translational research

## Abstract

More widespread use of positron emission tomography (PET) imaging is limited by its high cost and radiation dose. Reductions in PET scan time or radiotracer dosage typically degrade diagnostic image quality (DIQ). Deep-learning-based reconstruction may improve DIQ, but such methods have not been clinically evaluated in a realistic multicenter, multivendor environment. In this study, we evaluated the performance and generalizability of a deep-learning-based image-quality enhancement algorithm applied to fourfold reduced-count whole-body PET in a realistic clinical oncologic imaging environment with multiple blinded readers, institutions, and scanner types. We demonstrate that the low-count-enhanced scans were noninferior to the standard scans in DIQ (*p* < 0.05) and overall diagnostic confidence (*p* < 0.001) independent of the underlying PET scanner used. Lesion detection for the low-count-enhanced scans had a high patient-level sensitivity of 0.94 (0.83–0.99) and specificity of 0.98 (0.95–0.99). Interscan kappa agreement of 0.85 was comparable to intrareader (0.88) and pairwise inter-reader agreements (maximum of 0.72). SUV quantification was comparable in the reference regions and lesions (lowest *p*-value=0.59) and had high correlation (lowest CCC = 0.94). Thus, we demonstrated that deep learning can be used to restore diagnostic image quality and maintain SUV accuracy for fourfold reduced-count PET scans, with interscan variations in lesion depiction, lower than intra- and interreader variations. This method generalized to an external validation set of clinical patients from multiple institutions and scanner types. Overall, this method may enable either dose or exam-duration reduction, increasing safety and lowering the cost of PET imaging.

## Introduction

Positron emission tomography (PET) imaging is used for a wide range of clinical indications, including detecting, staging, and restaging tumors, dementia, and epilepsy, despite its relatively high cost and its use of radioactivity^1^. PET images are obtained by injecting patients with a standardized dose of a radiopharmaceutical (e.g., 18F-fluorodeoxyglucose [FDG]). The image quality is proportional to the number of coincidence events in the PET detector following radiopharmaceutical positron annihilation. Using the PET images, a quantitative standardized uptake value (SUV) can be calculated by normalizing the radiotracer uptake with its dosage and patient weight or lean body mass^2,3^. The maximum SUV (SUV_max_) is widely used as a semiquantitative measure of the tumor glucose metabolism^4^.

PET is commonly used in conjunction with computed tomography (CT) imaging to provide attenuation correction and anatomic localization. However, whole-body PET/CT scans can require upward of 30 min of table time, depending on the field of view and patient’s height. This can cause patient discomfort and anxiety, and may potentially lead to motion artifacts that degrade image quality and SUV quantitation^5,6^. Additionally, exposure to radiation during diagnostic imaging can increase cancer risk, especially in pediatric populations^7,8^, and raises concerns about exposure to technologists^9^. While such concerns make scanning with reduced bed times or lower radiotracer dose appealing, both actions lower detection of PET annihilation events, which reduces image quality and SUV accuracy. Consequently, reducing PET scan durations or using lower radiotracer dose without reducing the diagnostic image quality (DIQ) or biasing SUV measurements would be clinically valuable and would enable a higher throughput of patients for diagnostic imaging^10^. Recently, with the looming burden of COVID-19, which necessitates additional cleaning and sanitization of PET scanners, increased scanning efficiency will limit the bottlenecks in PET imaging for maintaining an adequate patient throughput.

Advances in deep learning and convolutional neural networks (CNNs) have presented an exciting opportunity in medicine, primarily for solving a variety of image-classification problems in radiology, pathology, and dermatology^11–14^. These methods have also been used to improve the quality of diagnostic radiology images for several imaging modalities^15,16^, and specifically, for synthesizing high-quality PET images from input images acquired either with a low radiotracer dosage or acquired over a shorter duration^17,18^. A handful of recent studies have demonstrated the denoising capabilities of CNNs to enhance PET image quality in small patient cohorts^19–21^. While most of the preliminary studies have focused on enhancing image quality, they have not been deployed in a realistic clinical setting and the impact on quantitative SUV measurements has not been robustly validated across vendors and reconstruction algorithms. Unlike other imaging modalities, to our knowledge, there have been no studies evaluating the clinical utility of low-count-enhanced PET scans^22,23^.

There are also many PET imaging vendors, each with unique hardware and software that can considerably affect overall image quality^24^. CNNs trained on a specific domain of inputs have consistently demonstrated limited generalizability when data from a different domain are presented to the network^25^. In medical imaging, it is becoming increasingly well-known that models developed in specific, narrow populations fail to generalize well to data from different institutions and scanner types^26,27^. Thus, a key question that has not been addressed with current low-count PET CNNs is that of *model generalizability*, i.e., how well can a model trained on one subset of data generalize to new unseen patients from different institutions and different scanners^28^.

To overcome the aforementioned challenges, in this study, we utilize deep learning to enhance the quality and maintain quantitative SUV accuracy of FDG PET scans acquired or simulated with 4x lower counts (i.e., equivalent to either four-fold faster or fourfold reduced dose). We evaluate both qualitative and quantitative performance in an external validation cohort drawn from cancer patients at multiple institutions scanned with a variety of PET scanner devices under typical clinical conditions. We hypothesize that deep-learning-enhanced fourfold reduced-count PET is noninferior to current standard-dose FDG PET for clinical evaluation purposes and is equivalent for SUV-based quantification of tumor burden.

## Results

### Low-count-enhanced images

**Fig. 1:**
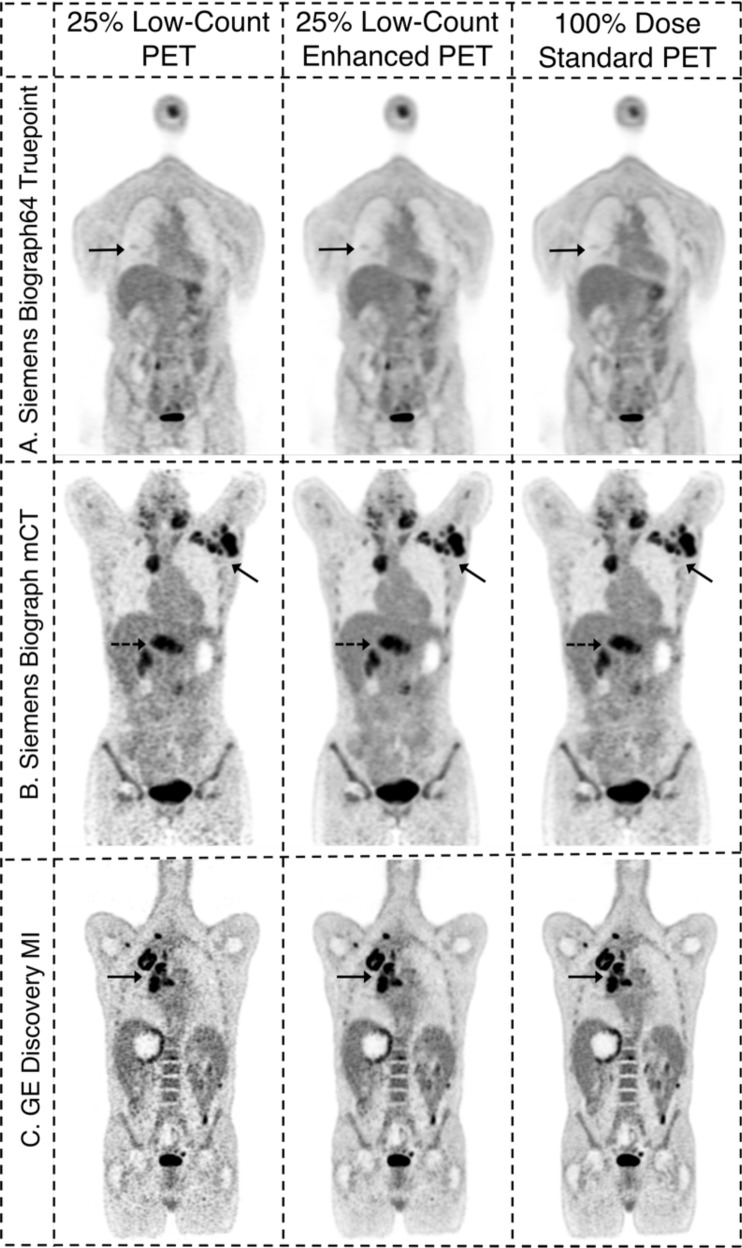
Example 25% low-count PET images, the 25% low-count-enhanced images, and the corresponding standard images for all three scanners for subjects with body mass index (BMI) under 30. **A** A 66-year old male with BMI of 27 scanned on a Siemens Biograph64 Truepoint for oropharyngeal cancer (solid arrow pointing to a distant lung metastasis). **B** A 34-year-old female with BMI of 20 scanned on a Siemens Biograph mCT for lymphoma (solid arrow pointing to metastatic lymph nodes and dashed arrow pointing to lesion in the spleen). **C** A 58-year-old male with BMI of 23 scanned on a GE Discovery MI with lung cancer (solid arrow). For all subjects, all low-count images appear considerably noisier compared with the low-count-enhanced and standard images. Note: SUV display scale is 0–7.

**Fig. 2:**
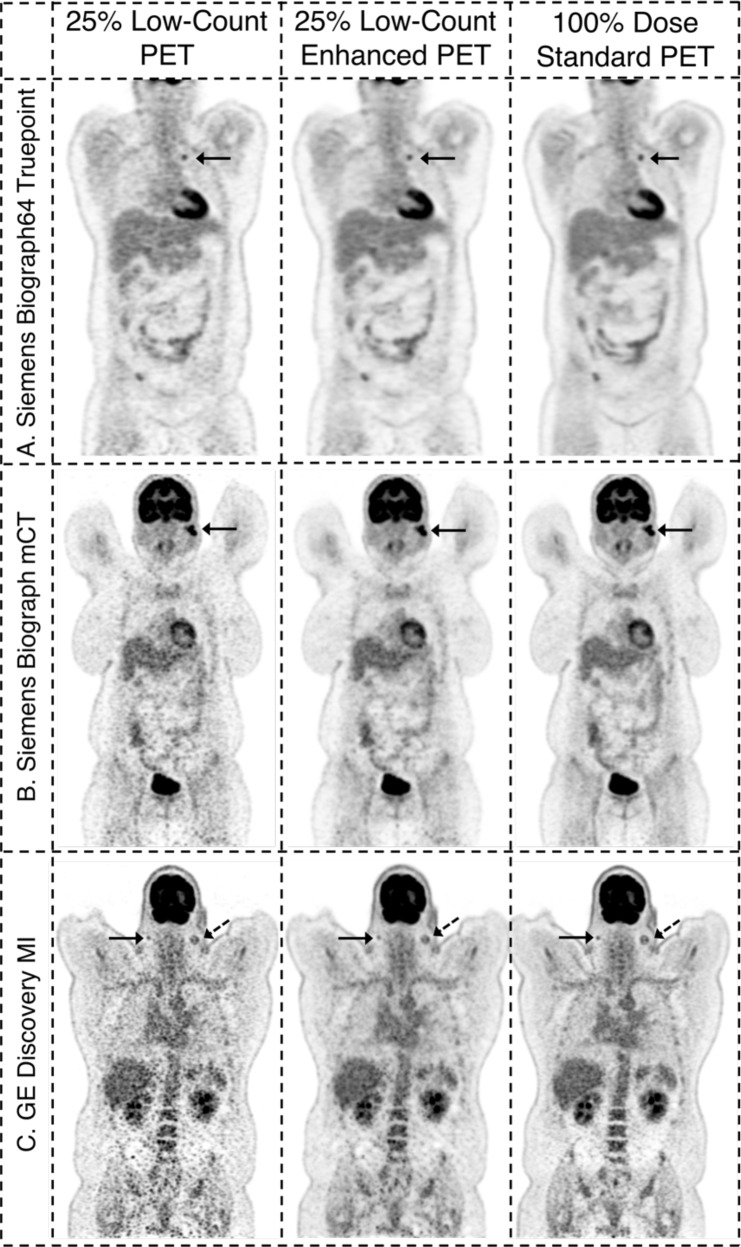
Example 25% low-count PET images, the 25% low-count-enhanced images, and the corresponding standard images for all three scanners for subjects with BMI over 30. **A** A 31-year-old female with BMI of 32 scanned on a Siemens Biograph64 Truepoint for colon cancer (solid arrow pointing to a distant lung metastasis). **B** A 38-year-old female with BMI of 36 scanned on a Siemens Biograph mCT for lymphoma (solid arrow pointing to parotid lymph nodes). **C** A 56-year-old male with BMI of 43 scanned on a GE Discovery MI with metastatic carcinoma of the head and neck (solid and dashed arrows pointing to separate metastatic lymph nodes). In the case of subjects with high BMI, the images are noisier for all scan types. The low-count-enhancement successfully denoises the low-count images and provides similar diagnostic conspicuity as the standard images. Note: SUV display scale is 0–7.

**Fig. 3:**
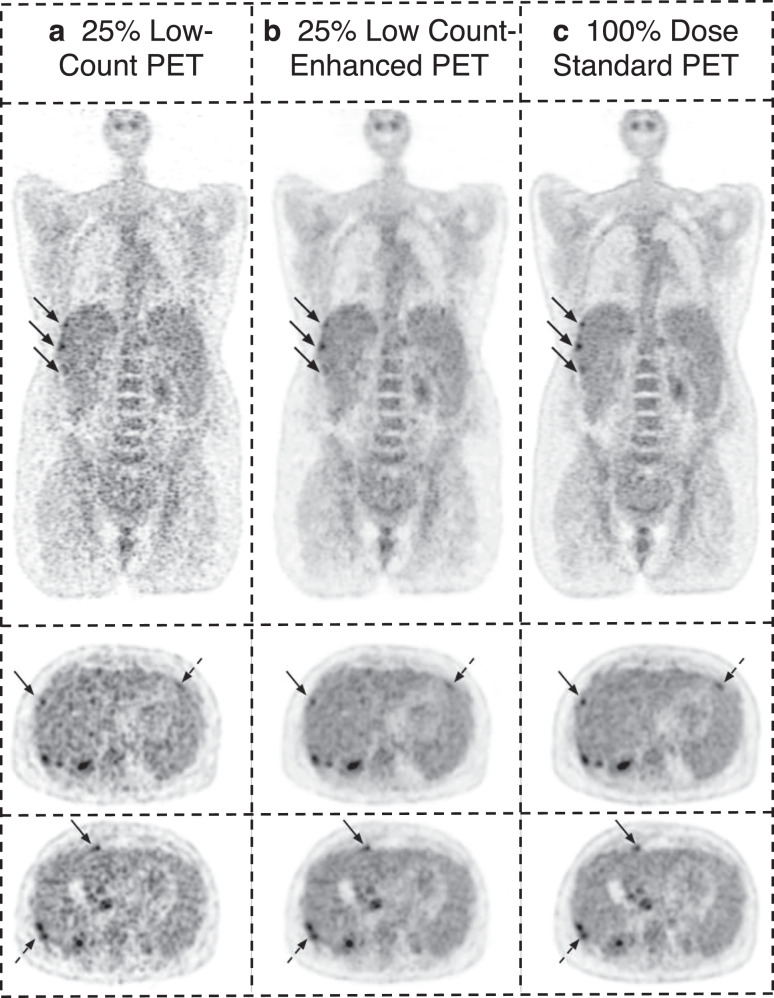
An example case showing the effectiveness of deep-learning enhancement of low-count PET in a 65-year-old female with BMI of 31 and an occult malignancy scanned on a GE Discovery MI scanner. A coronal slice and two axial slices through the liver are displayed for **A** 25% low-count, **B** 25% low-count-enhanced, and **C** standard PET images. The low-count PET images are noisy and lesions (arrows) are obscured by noise. The low-count-enhanced images are effectively denoised by the low-count-enhancement algorithm and the lesion conspicuity is similar to standard PET images. Note: SUV display scale is 0–7.

Representative whole-body PET images for the 25% low-count images, the 25% low-count-enhanced images using the CNN, and the corresponding 100% dose-standard images for all three scanners used in this study are shown in Figs. 1–3. Figure 1 shows three different patients all with a BMI of less than 30, with oropharyngeal cancer (Fig. 1a), lymphoma (Fig. 1b), and lung cancer (Fig. 1c). Similar images are shown for subjects with BMI over 30, with colon cancer (Fig. 2a), lymphoma (Fig. 2b), and metastatic carcinoma of the head and neck (Fig. 2c). The effectiveness of the deep-learning enhancement technique in improving image quality and lesion conspicuity compared with the low-count scans is shown in Fig. 3. Images from an example subject with a stump were also successfully enhanced using the enhancement model without creating any artifacts, despite the images being out-of-distribution compared with the training data (Supplementary Fig. 1).

### Image quality assessment

**Fig. 4:**
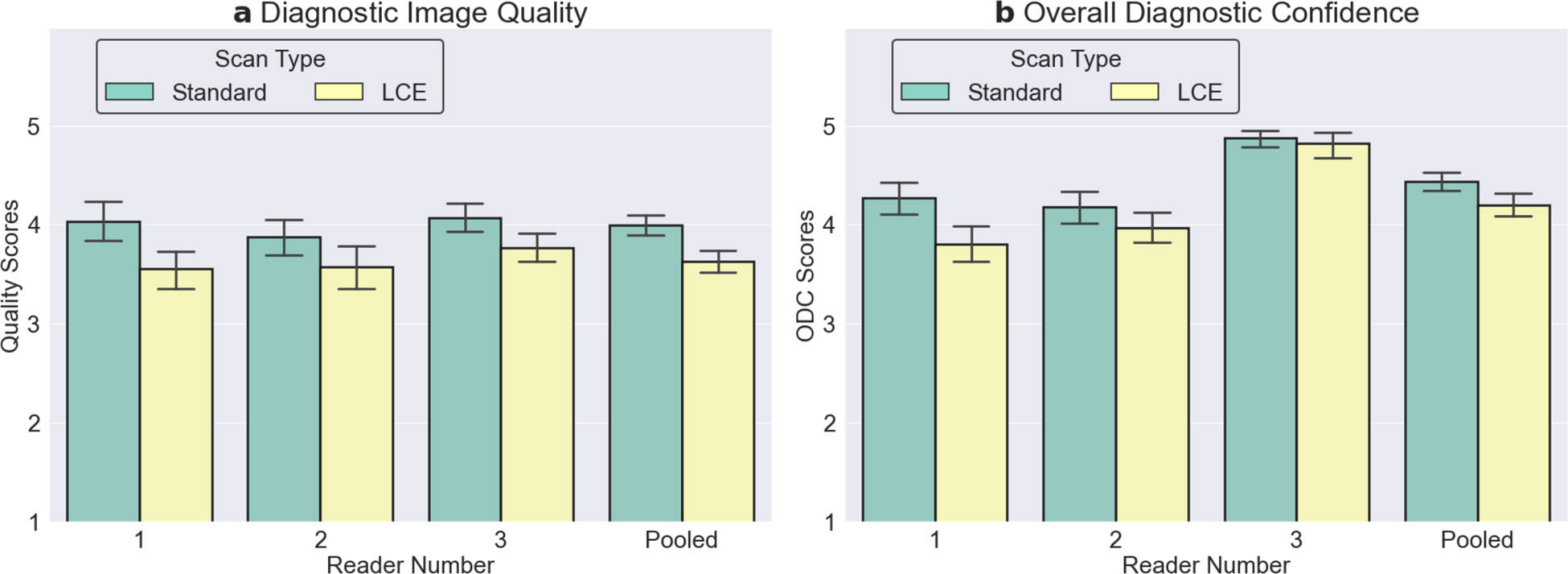
Diagnostic image quality (DIQ) and overall diagnostic confidence (ODC) scores (mean ± standard deviation) for the standard and low-count-enhanced (LCE) PET scans. The dashed line indicates clinically acceptable DIQ and ODC.

The DIQ ratings for the standard and low-count deep-learning enhanced scans were 4.0 ± 0.7, and 3.7 ± 0.7 respectively, while the ODC ratings were 4.5 ± 0.6 and 4.2 ± 0.8, respectively. DIQ and ODC values assigned by all three readers (Fig. 4) were comparable between the standard and low-count-enhanced PET scans, and were consistently higher than the minimum diagnostically acceptable quality (score of 3). Both the overall DIQ (*p* = 0.77) and ODC (*p* = 0.44) scores did not significantly vary as a function of the scanner used, but they did vary as a function of reader for both DIQ and ODC (both *p* < 0.01). The reader-based overall score heterogeneity was a random variable and was accounted for in the noninferiority analysis using the RMSE of the model. The interaction term between the imaging method (low-count-only and low-count-enhanced scans) and the scanners was nonsignificant for both DIQ (*p* = 0.59) and ODC (*p* = 0.28), showing that the difference in scores and image-quality enhancement between the low-count-only and low-count-enhanced images did not depend on the underlying scanner used.

The point estimate for differences between the standard and low-count-enhanced methods and their 95% confidence intervals (CI) for DIQ was 0.35 (95% CI of 0.21-0.49) and for ODC was 0.25 (95% CI of 0.14–0.36), with the respective *p*-values of 0.02 and <0.001 (Supplementary Figure 2). The results demonstrated the noninferiority for both DIQ and ODC with the proposed technique. The standard deviations for ODC and DIQ differences between the two imaging methods were 0.67 and 0.81, respectively, both of which were within the maximum tolerable standard deviation for a sample size of 50 patients^29^.

All standard PET scans maintained a DIQ and ODC of three (clinically acceptable) or higher. In total, 8/180 (3.9%) and 2/180 (1.1%) of the reads of the low-count-enhanced scans were scored as 2 (poor) for DIQ and ODC, respectively. None were scored as 1 (nondiagnostic). On an individual reader basis, this was distributed in the following manner: DIQ (two cases for reader 1, five cases for reader 2, and one case for reader 3) and ODC (one case each for readers 1 and 3).

### Repeatability assessment

ICC values for interreader repeatability of DIQ and ODC were 0.25 and 0.33, respectively. The interreader rating confusion matrices (Supplementary Tables 1 and 2) demonstrated that although the readers had different proclivities in providing scores of 3, 4, and 5 (Supplementary Fig. 3), all readers consistently graded the standard scans as similar to the low-count-enhanced ones. For the 10 cases that were read twice, there were no significant intrareader variations for any reader for both DIQ and ODC (DIQ p-values of 0.62, 0.12, and 0.02 and ODC *p*-values of 0.71, 0.05, and 0.32 for readers 1–3, respectively, with a Holm–Bonferroni-adjusted critical *p*-value of 0.017). Confusion matrices for intrareader repeatability are provided in Supplementary Tables 3 and 4.

### Lesion detection

A combined total of 491 hypermetabolic lesions were detected in 92 total standard scans read by the three readers. There were no hypermetabolic lesions detected in the brain. The number of hypermetabolic lesions detected on the standard and low-count-enhanced scans were not statistically different (*p* = 0.30) and there was a strong correlation in the number of lesions detected on the two scans (CCC 0.88, more detail in Table 1). There were no systematic biases in up-staging or downstaging subjects based on the number of lesions detected on the low-count-enhanced scans compared with the standard scans for all organs evaluated (Table 1). An example case of a subject receiving a higher DIQ score on the LCE scan than the standard scan in the case of a lung nodule is shown in Supplementary Fig. 4.

**Table 1:** Patient-level sensitivity and specificity with 95% confidence intervals (CI) along with lesion-level prevalence of hypermetabolic lesions and concordance correlation coefficient (CCC) as detected by the three readers (pooled) between the low-count-enhanced scans compared to the standard scans.

Organ	Sensitivity:	Specificity	CCC (All Lesions)	MW U-Test *P*-Value^a^ (All Lesions)	Homogeneity *P*-Value^b^
Bone	1.00 7/7	0.98 (0.88–1.00) 42/43	0.92	0.77	0.93
Lymph nodes	0.96 (0.80–1.00) 24/25	1.00 25/25	0.85	0.49	0.75
Liver	1.00 3/3	1.00 47/47	0.96	0.99	1.00
Lung	0.89 (0.52–1.00) 8/9	0.98 (0.87–1.00) 40/41	0.59	0.98	0.92
Muscle	0.75 (0.19–0.99) 3/4	0.98 (0.88–1.00) 45/46	0.77	0.93	0.99
Spleen	1.00 2/2	0.96 (0.86–0.99) 46/48	0.17	0.77	0.98
Overall	0.94 (0.83–0.99) 47/50	0.98 (0.95–0.99) 245/250	0.88	0.97	0.88

^a^Mann–Whitney (MW) U-tests evaluated whether the number of lesions detected using the two sets of images were statistically different.

^b^Homogeneity test evaluated whether there was a systematic bias in up-staging or down-staging patients.

The overall patient-level sensitivity and specificity for detecting lesions on the low-count-enhanced scan compared with the standard scan was 0.94 (0.83–0.99) and 0.98 (0.95–0.99) (Table 1). There were no significant differences in the total and organwise number of hypermetabolic lesions detected between the repeated reads pooled across all readers (lowest *p*-value = 0.48), indicating high repeatability of the readings. Example images from a subject with subtle liver lesions and a noisy low-count scan depicted that the low-count-enhancement technique successfully maintained high lesion conspicuity, despite starting with noisy images (Supplementary Fig. 5). Overall, there were eight instances of false positives or false negatives across 50 patients and six tissue types as assessed by the three readers. The distribution of these deviations did not follow any specific pattern as a function of the tissue of institution—bone (one false positive from Institution B), lymph nodes (one false negative from Institution C), lung (one false positive and false negative each from Institution A), spleen (two false positives from Institution B), and muscle (one false positive and false negative each from Institution C).

The Cohen’s kappa value of 0.85 (95% confidence interval 0.81–0.90) for inter-scan agreement between the lesions detected by the standard and low-count-enhanced scan was nearly identical to the kappa of 0.88 (0.82–0.95) for intrareader agreement between repeated readings for the same patient by the same reader. Similarly, the agreement between the low-count-enhanced and standard scans was higher than that for inter-reader agreement for all pairs of readers: Cohen’s kappa for readers 1 and 2 = 0.58 (0.50–0.66); readers 1 and 3 = 0.69 (0.62–0.76); readers 2 and 3 = 0.72 (0.65–0.79) (Table 2).

**Table 2:** Cohen’s kappa comparing interreader lesion detection agreement between pairs of readers, intrareader agreement between repeated reads of the same subject, and interscan agreement between the standard and low-count-enhanced scans, stratified by the type of scans and readers.

Interreader agreement (*N* *=* 120)	Reader pairs		
	1 and 2	1 and 3	2 and 3
Low-Count-Enhanced	0.55 (0.42–0.67)	0.66 (0.55–0.77)	0.72 (0.61-0.83)
Standard	0.65 (0.53–0.76)	0.71 (0.61–0.81)	0.75 (0.65-0.85)
Overall	0.60 (0.51–0.68)	0.69 (0.61–0.76)	0.73 (0.66-0.81)
Intrareader Agreement (*N* *=* 20)	Reader number		
	1	2	3
Low-Count-Enhanced	0.74 (0.5–0.98)	0.74 (0.46–1.0)	0.95 (0.85-1.0)
Standard	1.0 (1.0–1.0)	0.84 (0.62–1.0)	0.84 (0.88-1.0)
Overall	0.88 (0.76–0.99)	0.79 (0.46–1.0)	0.95 (0.88-1.0)
Total Intrareader	0.88 (0.82–0.95)		
Interscan Agreement (*N* *=* 60)	Scan-pairs		
	Low-count-enhanced and standard		
Reader 1	0.84 (0.76–0.91)		
Reader 2	0.85 (0.76–0.93)		
Reader 3	0.88 (0.81–0.95)		
Overall	0.85 (0.81–0.90)		

### SUV equivalence

**Fig. 5:**
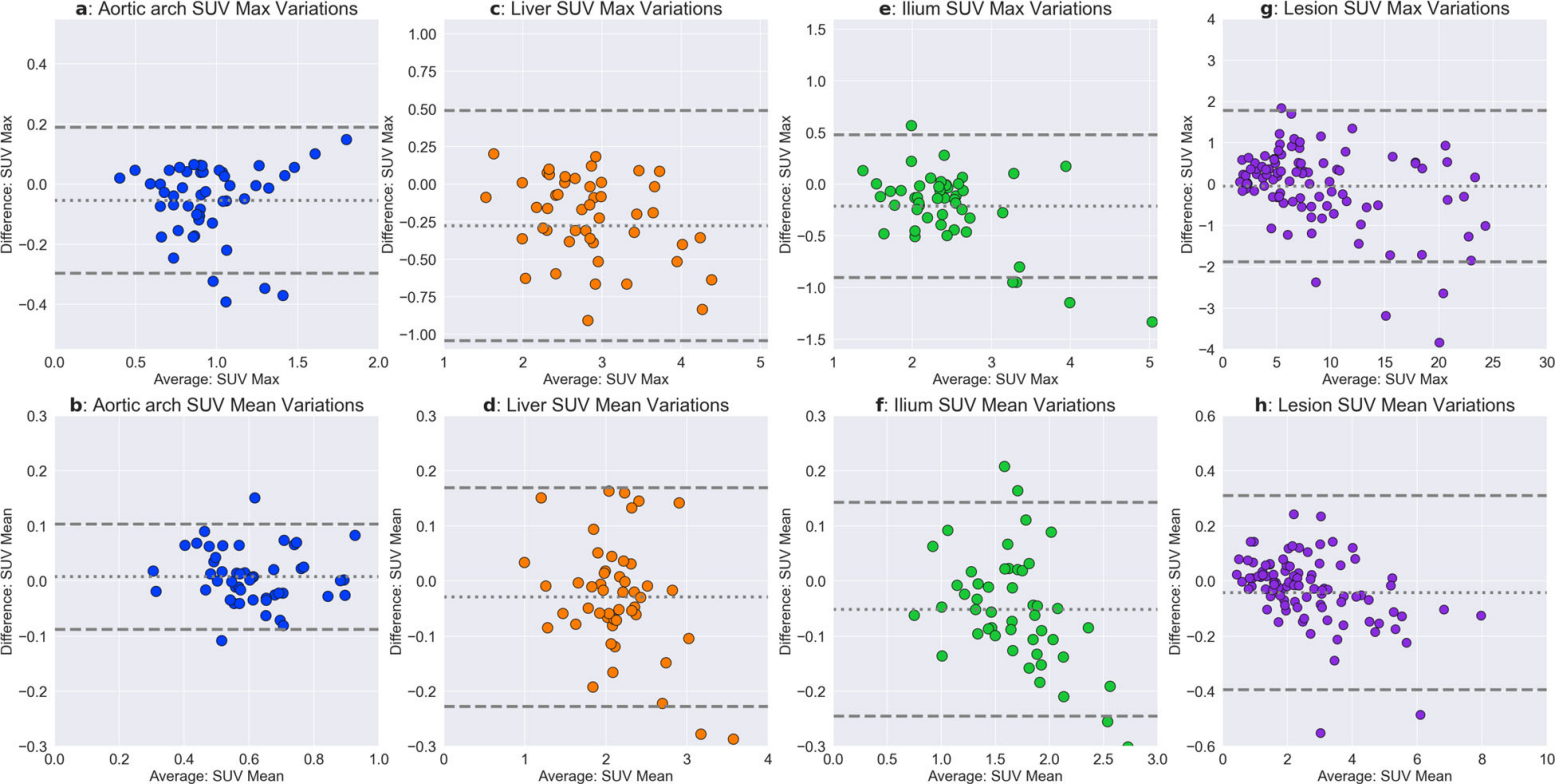
Quantitative SUV accuracy evaluation. Bland–Altman and regression plots showing the variations between in the SUV_mean_ for the aortic blood pool (**A**, **B**), liver (**C**, **D**), and right gluteus muscle (**E**, **F**) between the full-dose standard PET scans and the 25% LCE scans, along with SUV_max_ variations between hypermetabolic lesions (**G**, **H**). The Bland–Altman bias (dotted line) is nearly 0 for all anatomic structures and the 95% limits of agreement (dashed lines) were considerably lower than the average Bland–Altman values. The regression plots demonstrate a strong correlation between the SUV metrics for the standard and LCE scans. No statistically significant differences were observed for any of the four anatomic structure SUV values between the two scans. The largest absolute differences in SUV_max_ are seen at very high levels that represent very highly metabolic regions.

In total, 99 hypermetabolic lesions were identified on the standard PET scans by the fourth reader. CCC and Bland–Altman plots for SUV_mean_ of aortic blood pool, liver, and right gluteus muscle reference regions and the SUV_max_ of the lesions showed minimal variation among the standard and low-count-enhanced scans (Fig. 5). The mean difference of the SUVs was approximately zero (ranging from −0.05 to 0.01 for the different regions), with tight 95% confidence limits of agreement (0.10–0.20 for the SUV_mean_ of the reference regions, and 1.8 for the SUV_max_ of the lesions) (Table 3). Correlation coefficients were very high between the two scans: aortic blood pool (CCC = 0.94), liver (CCC = 0.98), gluteus muscle (CCC = 0.96), and lesions (CCC = 0.99). No statistically significant differences were found for SUV values for the aortic blood pool (*p* = 0.91), liver (*p* = 0.80), gluteus muscle (*p* = 0.59), and lesions (*p* = 0.79). Using an SUV_max_ threshold of 2.5, the sensitivity and specificity of lesion detection on the low-count-enhanced scans was 0.98 (0.93–1.00) and 1.0 (1.0–1.0). For the determination of a lesion as a function of its SUV, two false negatives occurred when the SUVmax values on the standard scans were 2.8 and 2.7, but on the low-count-enhanced scans were 2.4 and 2.1, respectively. Both cases arose from different subjects from Institution C.

**Table 3:** Comparison of quantitative SUV metrics between the standard and low-count-enhanced PET scans. SUV_mean_ was computed for the aortic blood pool, gluteus muscle, and liver, while SUV_max_ was computed for hypermetabolic lesions.

Organ	Count	Concordance	RMSE-CV^a^	Bias	95% CI Limits of Agreement	MW U-Test *P*-Value
Aortic Blood Pool	50	0.93	4.3%	0.01	±0.10	0.91
Gluteus Muscle	50	0.96	3.1%	−0.05	±0.19	0.59
Liver	50	0.98	2.4%	−0.03	±0.20	0.80
Lesions	99	0.99	5.7%	−0.05	±1.83	0.79

^a^Abbreviations: root-mean-square error coefficient of variation (RMSE-CV).

## Discussion

In this study, we demonstrated noninferiority of deep learning to enhance noisy PET images acquired with 4-times fewer counts for clinical purposes in a blinded, multicenter study. Quantitative accuracy for SUV measurements and depiction of hypermetabolic lesions were also maintained. Compared with other low-count PET-enhancement methods using CNNs, the findings in this study were demonstrated in a clinically relevant, external validation cohort that consisted of subjects with varying pathological findings that were assessed by board-certified nuclear medicine physicians. Moreover, the patients were scanned at different institutions on different vendor hardware that used different image-reconstruction algorithms, none of which was used to train the CNN model. We therefore conclude that the low-count-enhancement algorithm enabled the acquisition of PET data four-times faster or at fourfold reduced dose while providing similar diagnostic information as a standard PET examination in a generalizable manner across PET vendors and scanner models.

Since faster and lower-dose scans lead to similar image quality in this regime, the method demonstrated could be used either to increase efficiency or to reduce dose^30,31^. Increased efficiency is beneficial, given the potential to reduce the cost of PET and enhance patient throughput by scanning more patients on a single scanner. Decreased bed times for PET imaging would also allow for additional duration for sanitization protocols in trying to mitigate the spread of COVID-19. It may also have beneficial effects for patients who cannot tolerate longer scans and are susceptible to motion artifacts, which further degrades images. The use of lower dose may be beneficial to populations in which secondary malignancies might occur, and particularly for pediatric patients who are more sensitive to radiation and may receive many PET scans over their lifetime^8^.

The images included in the nuclear-medicine physician-reader study to evaluate lesion conspicuity, image quality, and diagnostic confidence consisted of the standard and LCE PET scans since low-count-only PET has previously demonstrated nondiagnostic image quality^31–33^. All three readers who analyzed the diagnostic utility of the two sets of PET scans consistently rated the low-count-enhanced scans as noninferior to the standard scans. These findings were consistent, despite the scans being acquired from PET/CT scanners of different vendor makes and models. The relative differences in ratings between the full-dose and low-count-enhanced scans were consistent across all three scanners, showing that the CNN was capable of consistently enhancing the low-count images independent of the scanner hardware and software. The intrareader repeatability on the blinded repeatability set of 10 subjects was also high, which indicated reader reliability in assessing the low-count-enhanced and standard scans. The three different readers in this study hailed from three different institutions with different PET scanner hardware and reconstruction algorithms. Consequently, intrareader variations in the assignment of DIQ and ODC scores may be reflective of their experience and comfort in interpreting different images with specific image quality created from different PET scanners.

The depiction of hypermetabolic lesions had a high sensitivity, specificity, and correlations between the two sets of images. Moreover, the combination of regression metrics such as CCC with classification metrics such as the presence/absence of lesions provided a good indication of the overall accuracy. In particular, the patient-level detection of lesions in the lymph nodes, a primary indication of cancer metastasis, was near-perfect between the low-count-enhanced images and the standard images. The prevalence rates for lesions in the muscle were low, with three out of the four lesions correctly being identified in the low-count-enhanced scans. In several instances with other subjects, the readers commonly reported diffuse regions of increased uptake as opposed to a focal uptake that could not be categorized as a lesion. Significantly, the variation observed in the lesion depiction between the standard and low-count-enhanced scans was comparable to the observed intra- and interreader variation. This suggests that the lesion conspicuity between the two scans was dependent on readers’ proclivities in addition to the images themselves, further demonstrating the noninferiority and lack of a systematic bias in the lesion-depiction capability of the low-count-enhancement technique.

While maintaining image quality is vital, PET is a quantitative technique, and thus it is essential to maintain SUV accuracy. We found high concordance for SUV_mean_ in the aortic blood pool, liver, and right gluteus muscle—organs typically used as internal references. Similarly, SUV_max_ was comparable for hypermetabolic lesions also. Maintaining comparable SUV quantitation between the low-count-enhanced and standard PET images is important to assess tumor avidity and response to therapy. Coupled with the high concordance in the depiction of lesions and the SUV quantification between the standard and low-count-enhanced scans, the proposed method maintained pixelwise accuracy and imagewise quality, two criteria important for prospective image-acquisition studies^34^. Overall, this shows that the proposed method can be used for accurate staging and prognostication.

The study has the following limitations. During the reader study for determining hypermetabolic lesions, the three blinded readers only indicated the organs of involvement but did not indicate specific lesions. Correlating the diagnostic performance of the low-count-enhanced images as a function of lesion, VOI volume may be beneficial for staging and prognostication. Additionally, the subjects that were included in this study underwent a whole-body PET/CT protocol. The performance of the deep-learning model for other types of studies (such as for neuroimaging) was not performed. Moreover, this study only included patients scanned with FDG, while this is by far the largest clinical use case for PET-CT; the use of the deep-learning enhancement with non-FDG radiotracers may have different performance dependent upon signal-to-noise ratios and the uptake dynamics and locations. Ethnicity information was also not collected in this initial proof-of-concept study; however, future work will be necessary to determine whether there exist systematic ethnicity-based biases in model performance.

In conclusion, we have evaluated the use of a deep-learning technique to enhance image quality of fourfold count-reduced PET images in a multicenter and multivendor study. The low-count-enhanced PET scans maintained image quality and SUV accuracy as assessed by nuclear-medicine physicians from three different institutions. The testing datasets used in this study included PET images from three institutions and three different PET-CT scanners, previously unseen by the CNN, demonstrating generalizability in an external validation cohort. The variations in lesion depiction between the standard and low-count-enhanced scans were lower than intra- and interreader variation. Thus, the proposed low-count-enhancement technique is promising to enable lower radiotracer dose and to improve the efficiency of diagnostic PET imaging.

## Methods

### Patient population

In total, 50 subjects from three separate hospitals (20 from Institution A, 10 from Institution B, and 20 from Institution C) referred for a whole-body FDG PET/CT examination (between September 2018 and April 2019) were included in this prospective study. All subjects were scanned with Institutional Review Board approval, informed consent, and Health Insurance Portability and Accountability Act compliance. IRB approvals were obtained from the University of Southern California and Oregon Health Sciences University, while a research ethics board (REB) approval was obtained from the University of Toronto. Consecutive adult, nonpregnant patients undergoing a standard skull-base to mid-thigh FDG PET-CT were eligible. The clinical indications for the studies were for cancer diagnoses, with full demographic information presented in Table 4. In total, 26 male patients (mean age: 58 ± 17 years, range:19-90 years) and 24 female patients (mean age: 58 ± 17 years, range: 26–85 years) undergoing PET imaging with a mean FDG dose of 12.0 ± 1.9 mCi were evaluated in this study.

**Table 4:** Demographics of subjects included in this study and the corresponding positron emission tomography (PET) scanner specifications for a PET/ CT (computed tomography) scanner with a whole-body fluorodeoxyglucose (FDG) protocol and a 75% reduction in bedtime.

Institution #	A	B	C
Number of patients	20	10	20
Age (years)	61 ± 17 Range: 19 − 80	50 ± 19 Range: 24 − 77	58 ± 16 Range: 26 − 90
Sex	9 M: 11 F	6 M: 4 F	11 M: 9 F
Body mass index	26.5 ± 4.0	23.5 ± 5.5	28.5 ± 6.3
Standard FDG dose (mCi)	13.0 ± 1.6	10.8 ± 2.2	11.6 ± 1.4
Scanner model	Siemens Biograph64 Truepoint	Siemens Biograph mCT	GE Discovery MI
Standard scan time	3 mins/bed	2 mins/bed	3 mins/bed
Average number of bed positions	7	7	7
Reconstruction technique^a^	OSEM + PSF 3i 21 s	OSEM + PSF + TOF 3i 21 s	OSEM + TOF + SharpIR 2i 24 s
Low-count methodology	2^nd^ Fast scan	List mode reconstruction of shorter duration	List mode reconstruction of shorter duration
Indications	Colon cancer (7) Lymphoma (4) Cholangiocarcinoma (1) Esophageal cancer (1) Leiomyosarcoma (1) Lung cancer (1) Melanoma (1) Renal cancer (1) Sarcoma (1) Spinal fusion (1) Unknown/Declined (1)	Lymphoma (5) Lung cancer (2) Esophageal cancer (1) Occult malignancy (1) Unknown/Declined (1)	Lymphoma (4) Esophageal cancer (2) Breast cancer (2) Occult malignancy (2) Chronic lymphocytic leukemia (1) Dedifferentiated Chondrosarcoma (1) Gastric cancer (1) Head & neck Cancer (1) Lung cancer (1) Melanoma (1) Neoplasm of unspecified etiology (1) Pancreatic Neuroendocrine tumor (1) Rectal cancer (1) Urethral cancer (1)

^a^*OSEM* ordered subset expectation maximization, *PSF* poin t spread function, *TOF* time of flight.

### Low-count PET enhancement

The FDG dose and the uptake time for the PET scans was based on the standard protocol at each institution (Siemens Biograph64 Truepoint with 3 min/bed acquisition at Institution A, Siemens Biograph mCT with 2 min/bed acquisition at Institution B, and GE Discovery MI with 3 mins/bed at Institution C). The details of the reconstructions, which did not vary between the standard and low-count scans, are shown in Table 4. Low-count scans were either captured from a separate scan with fourfold shorter bed durations performed immediately following the standard scan (Institution A) or created by reconstructing shorter bed durations from the standard scan using list-mode data (Institutions B and C), which have shown image-quality equivalence previously^31,35^. An FDA-cleared, commercially available software product (SubtlePET, Subtle Medical, Menlo Park, CA) was used to enhance the low-count scans. This software uses a 2.5D encoder–decoder U-Net deep convolutional neural network (CNN) to perform denoising, and was trained on pairs of low- and high-count PET studies^36^. None of the subjects nor the institutions in this study contributed to the training of the deep network, making this a true external validation test.

### Reader study for low-count-enhanced scans

Previous studies have demonstrated considerably reduced diagnostic conspicuity for hypermetabolic lesions and biased SUV measurements for fourfold low-count PET^31–33^. Consequently, the goal of this reader study was to investigate whether the low-count-enhanced PET scans using deep learning were noninferior to the standard of care. Moreover, such an approach would also lower reader fatigue and case-memorization effects that may arise if readers assessed the standard of care scans, low-count scans, and low-count-enhanced scans.

For the reader study, three board-certified nuclear-medicine physicians from three separate academic institutions evaluated the efficacy of the low-count-enhanced PET scans (G.D., E.M., and J.H. with 7, 12, and 22 years of experience). The readers evaluated two sets of PET scans per patient. One set consisted of the original 100% full-count PET scans (“standard”) and one set consisted of 25% low-count PET scans enhanced using the CNN (“low-count-enhanced”). The readers were blinded to the scan type (standard or low-count-enhanced). In addition, 10 patients were randomly chosen from the 50 original patients (4, 3, and 3 from each institution) and represented to the readers for a duplicate read to evaluate intrareader repeatability. None of the readers were made aware of this subject repetition. Overall, the 3 readers read 120 scans each (60 standard scans and 60 low-count-enhanced scans), which led to a total of 360 individual assessments.

All readers viewed the PET scans and the corresponding CT series using MIM Encore (MIM Software Inc., Cleveland, Ohio) to replicate their standard clinical reading environment. For each subject, the readers were allowed to generate multiplanar reformats and maximum-intensity projections (MIPs) as desired. After reviewing all images in the PET series, the readers were asked to score the diagnostic image quality (DIQ) on a 5-point Likert scale (1 = nondiagnostic, 2 = poor, 3 = acceptable, 4 = good, 5 = excellent image quality). The readers were also asked to indicate the number of hypermetabolic lesions depicted in the PET scan in the following organs: brain, lymph nodes, lung, liver, spleen, bone, and muscle. If more than five lesions were found in any region, the readers were instructed to report 5+ lesions. The readers were also asked to provide their overall diagnostic confidence (ODC) in interpreting the images on a Likert scale of 1–5 (1 = none, 2 = poor, 3 = acceptable, 4 = good, 5 = excellent diagnostic confidence).

A separate fourth reader (S.S., a board-certified nuclear-medicine physician with 13 years of experience) separately reviewed the standard PET scans from the 50 subjects. This reader drew volumes of interest (VOI) in the several reference regions (aortic blood pool, liver, and right gluteus muscle) and up to five abnormal lesions. For the liver, a 3-cm-diameter VOI was placed in segment VII. VOIs for up to five lesions were placed on a mix of higher- and lower-uptake lesions, to account for those with lower SUV_max_. All VOIs were subsequently copied from the standard scans to the low-count-enhanced scans. The SUV_mean_ of the VOIs was evaluated for the aortic blood pool, liver, and the right gluteus muscle as internal organ reference regions^37–39^, while the SUV_max_ was evaluated for the hypermetabolic lesions on the standard and low-count-enhanced scans.

### Statistical analysis

DIQ and ODC for both the low-count-enhanced and standard images were analyzed using a general linear model taking into account the following sources of variation: the two different imaging methods (within subjects), the three scanners, the three readers, 50 subjects, and interaction between the imaging method and scanners. We sought to investigate both the possibility of scanner variability and the presence of heterogeneity in the difference between both imaging methods due to an interaction with the underlying scanner model. The residual standard deviation (root mean square error), with its degrees of freedom, was used as an estimate of the variability of the scores. The model for noninferiority was chosen once the sources of noise were discarded. It was used to compute 95% confidence intervals for the difference between the means of the standard and low-count-enhanced methods, and the test of noninferiority, using a noninferiority margin of 0.5 points on the DIQ and ODC Likert scales. The noninferiority margin was chosen as a midpoint between two consecutive points on the 1–5 Likert scale and using prior qualitative reader studies for PET image quality assessment^40^. For a significance level of 5% and a power of 90%, a noninferiority limit of 0.5 for a sample size of 50 patients would allow the standard deviation of the DIQ and ODC score differences between methods to be as large as 0.85^29^.

Interreader variation for DIQ metrics was assessed using an interclass correlation coefficient (ICC). Statistical significance for intrareader variation between the first and second reads was tested using Wilcoxon signed-rank tests for comparing the paired DIQ, ODC, and number of hypermetabolic lesions values in the 10 repeated cases. The number of instances where the low-count-enhanced scans did not meet a DIQ or ODC of three or higher (clinically acceptable) were tallied.

Variations between the number of hypermetabolic lesions detected by the readers between the sets of scans were evaluated using Mann–Whitney U-tests and quantified using concordance correlation coefficients (CCC). For quantitative analysis, any scans marked with 5+ lesions were treated as a scan with six lesions. A homogeneity test was performed using contingency tables using the low-count-enhanced and standard scans to assess for any systematic bias to up-stage or down-stage patients based on lesion count.

The sensitivity and specificity of the low-count-enhanced PET scans to depict lesions was evaluated at the patient level, with respect to the lesions depicted in the standard scans. The number of lesions depicted by the readers was set at a clinically relevant threshold value of one (one class for zero lesions and another class for 1+ lesions). To minimize the noise introduced by intrareader and inter-reader variation, a lesion in a subject was considered positive if it was detected by a majority of the readers (2 out of 3). To compare the variability of lesion detection between the standard and low-count-enhanced scans versus the inherent repeatability of the readers themselves between successive reads for the same patients, Cohen’s kappa was computed to compare *interscan* agreement (standard versus low-count-enhanced) and *intrareader* agreement between reads (reading 1 versus 2 for the duplicated cases). Additionally, *interreader* agreement and Cohen’s kappa was also computed between lesions depicted by pairs of readers (reader pairs consisting of readers 1 and 2, readers 1 and 3, and readers 2 and 3)

To evaluate equivalence for quantitative SUV between the standard and low-count-enhanced scans, the CCC and Bland–Altman plots were generated for comparing SUV_max_ and SUV_mean_ for lesions and reference regions, respectively. Additionally, Mann–Whitney U-tests were used to compare systematic biases between the two scans. Finally, sensitivity and specificity analysis was performed between the standard and low-count-enhanced scans for identifying lesions above or below an SUV_max_ threshold of 2.5^41^.

Overall, measures of noninferiority of image quality for the low-count-enhanced images as assessed by the DIQ and ODC metrics were the primary outcomes of this study. The assessment of nonstatistically significant SUV and lesion-detection metrics were the secondary outcomes of this study. All statistical analysis was performed using Python (version 3.6.7) using the NumPy (version 1.16) and SciPy (version 1.3) libraries. All plotting of figures’ graphical data was performed using the Python matplotlib (version 3.1) and seaborn (version 0.8.1) libraries. All statistical significance levels were set to an *α*=0.05 with a Holm–Bonferroni correction to adjust for multiple comparisons between multiple readers or scanner types, where necessary.

### Reporting summary

Further information on research design is available in the Nature Research Reporting Summary linked to this article.

## Supplementary information

Supplementary Information

Reporting Summary

## Data Availability

The data from this study are not publicly available in accordance to institutional requirements governing human subject privacy considerations. The data may be made available from the authors upon reasonable request subject to permission and approval from the corresponding organizations and institutional review boards.

## Code availability

The code may be made available from the authors upon reasonable request subject to permission and approval from the corresponding organizations and institutional review boards.
