# Author Correction: Low-count whole-body PET with deep learning in a multicenter and externally validated study

**DOI:** 10.1038/s41746-021-00512-6

**Published:** 2021-09-14

**Authors:** Akshay S. Chaudhari, Erik Mittra, Guido A. Davidzon, Praveen Gulaka, Harsh Gandhi, Adam Brown, Tao Zhang, Shyam Srinivas, Enhao Gong, Greg Zaharchuk, Hossein Jadvar

**Affiliations:** 1 Department of Radiology, Stanford University, Palo Alto, CA USA; 2 Department of Biomedical Data Science, Stanford University, Stanford, CA USA; 3 Subtle Medical, Menlo Park, CA USA; 4 Division of Diagnostic Radiology, Oregon Health & Science University, Portland, OR USA; 5 Department of Radiology, University of Pittsburgh Medical Center, Pittsburgh, PA USA; 6 Department of Radiology, University of Southern California, Los Angeles, CA USA

**Keywords:** Cancer imaging, Radionuclide imaging, Translational research

Correction to: *npj Digital Medicine* 10.1038/s41746-021-00497-2, published online 23 August 2021

The original version of this Article contained errors in the location of the arrows in Figures 1A, 1B, 2A, and 2B. The displayed image slice from the same patient has also been updated for Figure 2B. Additionally, the labels for Figures 1 and 2 have been corrected to “Low-Count” as stated in the figure legends. These have now been corrected in the PDF and HTML versions of the Article.

