# Supplementary material for: Low-count whole-body PET with deep learning in a multicenter and externally validated study
Source: NPJ Digit Med. 2021 Aug 23;4:127. doi: 10.1038/s41746-021-00497-2 (PMC8382711; doi:10.1038/s41746-021-00497-2)
Supplement: Supplementary file 1 — Supplementary Information [file 41746_2021_497_MOESM1_ESM.pdf]

Supplementary Table 1: Confusion matrices for the diagnostic image quality (DIQ) scoring for the three readers.

**Reader 1**		**Low-count-enhanced**			
**Standard**	**Score**	**2**	**3**	**4**	**5**
	**2**	0	0	0	0
	**3**	1	10	2	1
	**4**	1	8	7	1
	**5**	0	3	9	7
**Reader 2**		**Low-count-enhanced**			
**Standard**	**Score**	**2**	**3**	**4**	**5**
	**2**	0	0	0	0
	**3**	0	10	4	0
	**4**	4	7	14	0
	**5**	0	1	6	4
**Reader 3**		**Low-count-enhanced**			
**Standard**	**Score**	**2**	**3**	**4**	**5**
	**2**	0	0	0	0
	**3**	1	1	1	0
	**4**	0	8	27	0
	**5**	0	0	9	3

Supplementary Table 2: Confusion matrices for the overall diagnostic confidence (ODC) scoring for the three readers.

**Reader 1**		**Low-count-enhanced**			
**Standard**	**Score**	**2**	**3**	**4**	**5**
	**2**	0	0	0	0
	**3**	1	2	2	0
	**4**	0	12	11	2
	**5**	0	1	10	9
**Reader 2**		**Low-count-enhanced**			
**Standard**	**Score**	**2**	**3**	**4**	**5**
	**2**	0	1	0	0
	**3**	0	0	1	0
	**4**	0	7	23	3
	**5**	0	2	6	7
**Reader 3**		**Low-count-enhanced**			
**Standard**	**Score**	**2**	**3**	**4**	**5**
	**2**	0	0	0	0
	**3**	0	0	0	0
	**4**	1	0	1	5
	**5**	0	1	5	37

Supplementary Table 3: Intra-reader variation between reads 1 and 2 for DIQ and ODC as a function of different readers. Abbreviations: Po. = Pooled across readers.

Diagnostic Image Quality

R1	Read #1				
Read #2		2	3	4	5
	2	0	0	0	0
	3	0	4	6	0
	4	0	3	2	2
	5	0	1	1	1

R2	Read #1				
Read #2		2	3	4	5
	2	0	0	0	0
	3	0	2	1	0
	4	0	7	3	4
	5	1	1	1	0

R3	Read #1				
Read #2		2	3	4	5
	2	0	0	0	0
	3	0	0	1	0
	4	0	6	10	0
	5	0	2	0	1

Overall Diagnostic Confidence

R1	Read #1				
Read #2		2	3	4	5
	2	0	0	0	0
	3	0	1	3	1
	4	0	0	9	2
	5	0	2	1	1

R2	Read #1				
Read #2		2	3	4	5
	2	0	0	0	0
	3	0	0	1	0
	4	0	3	9	1
	5	0	1	4	1

R3	Read #1				
Read #2		2	3	4	5
	2	0	0	0	0
	3	0	0	0	0
	4	0	0	0	3
	5	0	0	1	16

**Po.**	**Read #1**				
**Read #2**		**2**	**3**	**4**	**5**
	**2**	0	0	0	0
	**3**	0	6	8	0
	**4**	0	16	15	6
	**5**	1	4	2	2

**Po.**	**Read #1**				
**Read #2**		**2**	**3**	**4**	**5**
	**2**	0	0	0	0
	**3**	0	1	4	1
	**4**	0	3	18	6
	**5**	0	3	6	18

1

Supplementary Table 5: Intra-reader variation between reads 1 and 2 for DIQ and ODC as a function of different institutions (institution A (IA), institution B (IB), and institution C (IC)). Abbreviations: Po. = Pooled across institutions.

Diagnostic Image Quality

IA	Read #1				
Read #2		2	3	4	5
	2	0	0	0	0
	3	0	2	1	0
	4	0	6	6	1
	5	0	0	1	1

IB	Read #1				
Read #2		2	3	4	5
	2	0	0	0	0
	3	0	1	2	0
	4	0	5	5	3
	5	0	2	0	0

IC	Read #1				
Read #2		2	3	4	5
	2	0	0	0	0
	3	0	3	5	0
	4	0	5	4	2
	5	1	2	1	1

Overall Diagnostic Confidence

IA	Read #1				
Read #2		2	3	4	5
	2	0	0	0	0
	3	0	0	0	0
	4	0	1	7	3
	5	0	1	1	5

IB	Read #1				
Read #2		2	3	4	5
	2	0	0	0	0
	3	0	1	0	1
	4	0	0	5	2
	5	0	0	3	6

IC	Read #1				
Read #2		2	3	4	5
	2	0	0	0	0
	3	0	0	4	0
	4	0	2	6	1
	5	0	2	2	7

Po.	Read #1				
		**2**	**3**	**4**	**5**
**Read #2**	**2**	0	0	0	0
	**3**	0	6	8	0
	**4**	0	16	15	6
	**5**	1	4	2	2

Po.	Read #1				
		**2**	**3**	**4**	**5**
**Read #2**	**2**	0	0	0	0
	**3**	0	1	4	1
	**4**	0	3	18	6
	**5**	0	3	6	18

1

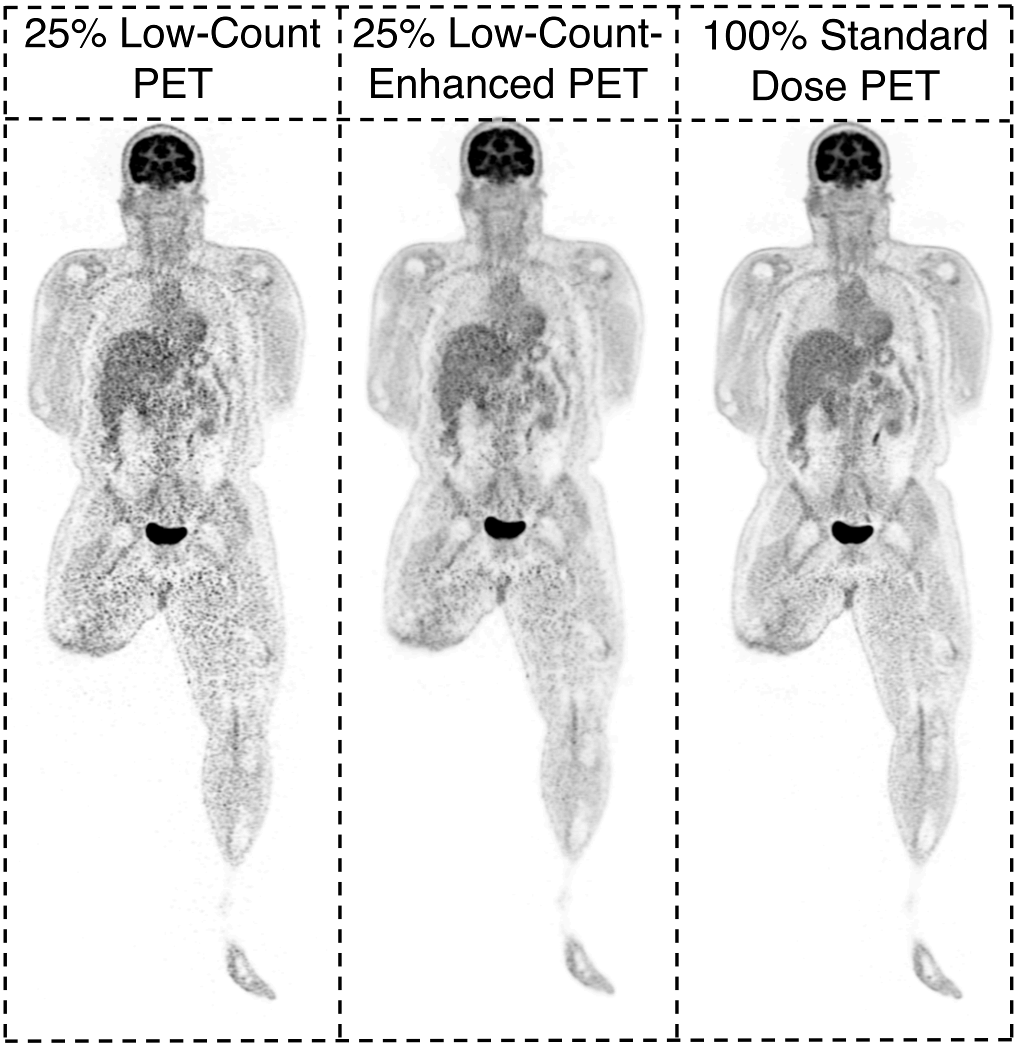

Supplementary Figure 1: Example 25% low-count images, 25% low-count-enhanced images, and the corresponding standard images for a 34-year old male with BMI of 28 scanned on a GE Discovery MI PET scanner. The subject had a right lower extremity stump and was an out-of-distribution input for the training corpus. Despite this, the low-count-enhancement algorithm successfully improved image quality of the low-count image without generating artifacts.

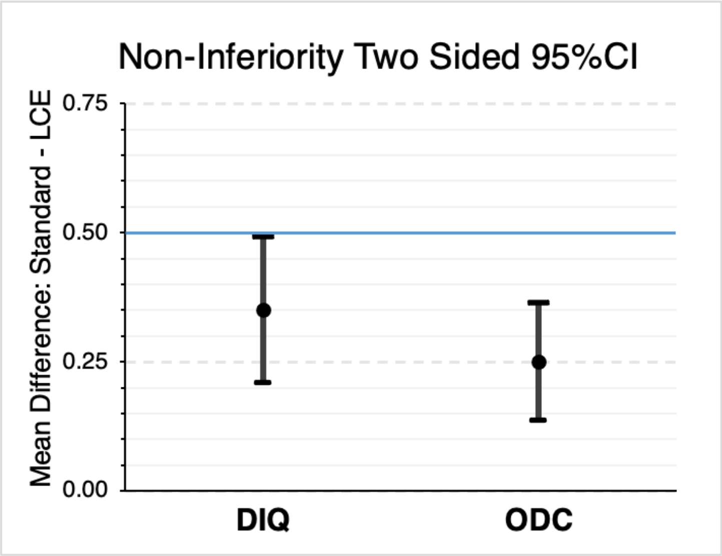

Supplementary Figure 2: Point estimates and corresponding 95% confidence intervals for demonstrating the non-inferiority of the low-count-enhancement (LCE) method for diagnostic image quality (DIQ) and overall diagnostic confidence (ODC) ratings for a threshold of 0.5 points (blue line) on the Likert scale.

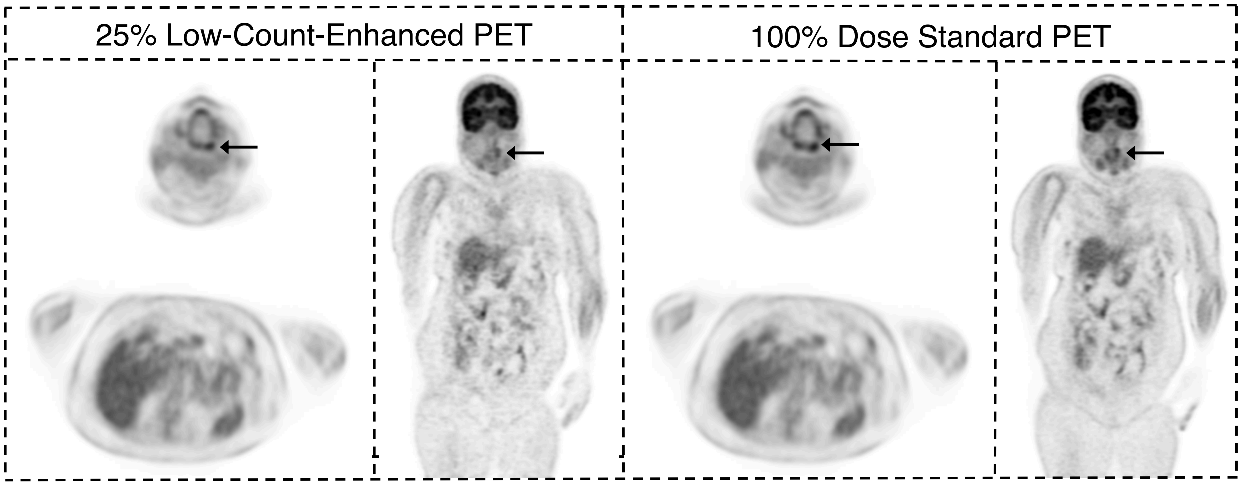

Supplementary Figure 3: Example 25% low-count-enhanced images and the corresponding standard images for a 31-year old female with BMI of 18 scanned on a Siemens Biograph64 TruePoint PET scanner. Axial slices through the neck and liver are seen, along with a coronal reformat. Increased metabolic activity is seen in the left tonsil (arrow). Despite the similar image quality, three different readers provided diagnostic image quality scores of 3, 4, and 5 for this patient's low-count-enhanced scan.

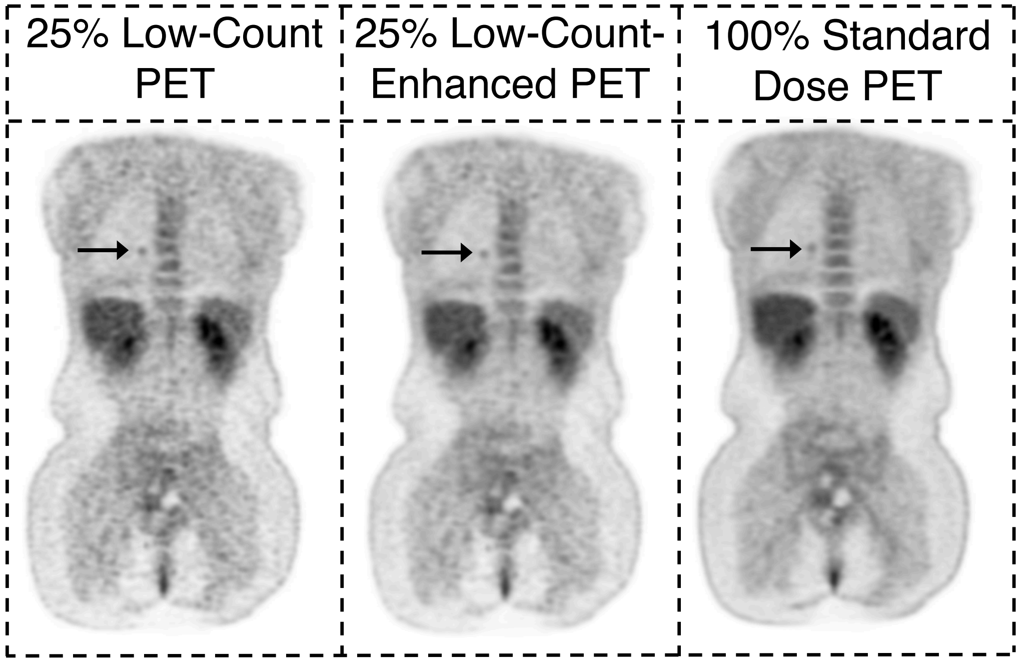

Supplementary Figure 4: Example 25% low-count images, 25% low-count-enhanced images, and the corresponding standard images for a 44-year old female with BMI of 20 scanned on a Siemens Biograph64 TruePoint PET scanner. A lung nodule is visible in the right lobe of the lung (arrow). Reader 1 rated the low-count-enhanced image with a DIQ of 5, while the standard-dose image was rated with a 3. Readers 2 and 3 rated both images with scores of 3 and 4, respectively.

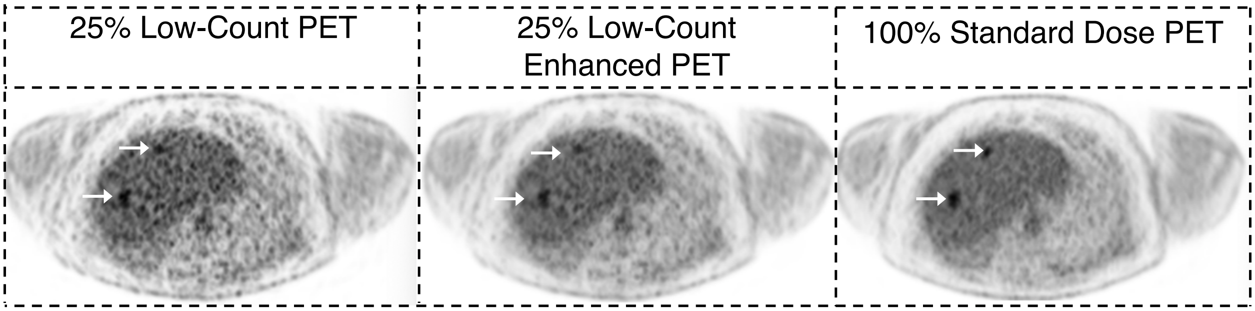

Supplementary Figure 5: Example 25% low-count images, 25% low-count-enhanced images, and the corresponding standard images for a 64-year old female with BMI of 24 scanned on a GE Discovery MI PET scanner. Two subtle lesions (arrows) are depicted in the liver with low lesion conspicuity due to the high background noise in the liver in the low-count image. Despite starting from a noisy image, the model was able to denoise the low-count-enhanced image without suppressing lesion conspicuity. Overall, both the low-count-enhanced and the standard dose images show improved conspicuity for the depiction of the same lesions.
