# Supplementary material for: Low-count whole-body PET with deep learning in a multicenter and externally validated study
Source: NPJ Digit Med. 2021 Aug 23;4:127. doi: 10.1038/s41746-021-00497-2 (PMC8382711; doi:10.1038/s41746-021-00497-2)
Supplement: Supplementary file 2 — Reporting Summary [file 41746_2021_497_MOESM2_ESM.pdf]

nature research

Corresponding author(s): Akshay Chaudhari

Last updated by author(s): 12-08-2020

Reporting Summary

Nature Research wishes to improve the reproducibility of the work that we publish. This form provides structure for consistency and transparency in reporting. For further information on Nature Research policies, see our Editorial Policies and the Editorial Policy Checklist.

Statistics

For all statistical analyses, confirm that the following items are present in the figure legend, table legend, main text, or Methods section.

n/a	Confirmed	
☐	☒	The exact sample size (n) for each experimental group/condition, given as a discrete number and unit of measurement
☐	☒	A statement on whether measurements were taken from distinct samples or whether the same sample was measured repeatedly
☐	☒	The statistical test(s) used AND whether they are one- or two-sided<br>*Only common tests should be described solely by name; describe more complex techniques in the Methods section.*
☒	☐	A description of all covariates tested
☐	☒	A description of any assumptions or corrections, such as tests of normality and adjustment for multiple comparisons
☐	☒	A full description of the statistical parameters including central tendency (e.g. means) or other basic estimates (e.g. regression coefficient) AND variation (e.g. standard deviation) or associated estimates of uncertainty (e.g. confidence intervals)
☐	☒	For null hypothesis testing, the test statistic (e.g. F, t, r) with confidence intervals, effect sizes, degrees of freedom and P value noted<br>*Give P values as exact values whenever suitable.*
☒	☐	For Bayesian analysis, information on the choice of priors and Markov chain Monte Carlo settings
☒	☐	For hierarchical and complex designs, identification of the appropriate level for tests and full reporting of outcomes
☒	☐	Estimates of effect sizes (e.g. Cohen's d, Pearson's r), indicating how they were calculated

Our web collection on statistics for biologists contains articles on many of the points above.

Software and code

Policy information about availability of computer code

Data collection	Poistron emission tomography data was collected on three different scanners: 1. Siemens Biograph64 Truepoint, 2. Siemens Biograph mCT, and 3. GE Discovery MI. Image quality enhancement was performed using an FDA approved software - SubtlePET (Subtle Medical, USA).
Data analysis	All statistical analysis was performed using Python (version 3.6.7) using the NumPy (version 1.16) and SciPy (version 1.3) libraries. All plotting of figures graphical data was performed using the Python matplotlib (version 3.1) and seaborn (version 0.8.1) libraries.

For manuscripts utilizing custom algorithms or software that are central to the research but not yet described in published literature, software must be made available to editors and reviewers. We strongly encourage code deposition in a community repository (e.g. GitHub). See the Nature Research guidelines for submitting code & software for further information.

Data

Policy information about availability of data

All manuscripts must include a data availability statement. This statement should provide the following information, where applicable:

- Accession codes, unique identifiers, or web links for publicly available datasets
- A list of figures that have associated raw data
- A description of any restrictions on data availability

The data from this study is not publicly available in accordance to institutional requirements governing human subject privacy considerations. However, the authors will try to provide the editors and reviewers the data for the purposes of evaluating this study.

Field-specific reporting

Please select the one below that is the best fit for your research. If you are not sure, read the appropriate sections before making your selection.

☒ Life sciences ☐ Behavioural & social sciences ☐ Ecological, evolutionary & environmental sciences

For a reference copy of the document with all sections, see nature.com/documents/nr-reporting-summary-flat.pdf

Life sciences study design

All studies must disclose on these points even when the disclosure is negative.

Sample size	No sample size calculation was performed. Consecutive subjects from three different hospitals were chosen to be included in this study. A total number of 50 unique subjects was agreed upon to ensure diversity of indications for imaging and to prevent reader fatigue during the qualitative evaluation of the images.
Data exclusions	No data were excluded.
Replication	Ten out of fifty subjects were asked to be re-reader by the nuclear medicine physicians to generate intra-reader replication. Statistical testing was performed on these repeated reads.
Randomization	All cases were presented to the readers in a completely random factor. This included randomization for the type of scan (standard dose or low-dose-enhanced), institution the subject was scanned at, repeated read or not, and indication.
Blinding	All readers were blinded to the type of scan being presented to them.

Reporting for specific materials, systems and methods

We require information from authors about some types of materials, experimental systems and methods used in many studies. Here, indicate whether each material, system or method listed is relevant to your study. If you are not sure if a list item applies to your research, read the appropriate section before selecting a response.

Materials & experimental systems

n/a	Involved in the study	
☒	☐	Antibodies
☒	☐	Eukaryotic cell lines
☒	☐	Palaeontology and archaeology
☒	☐	Animals and other organisms
☐	☒	Human research participants
☒	☐	Clinical data
☒	☐	Dual use research of concern

Methods

n/a	Involved in the study	
☒	☐	ChIP-seq
☒	☐	Flow cytometry
☒	☐	MRI-based neuroimaging

Human research participants

Policy information about studies involving human research participants

Population characteristics	50 subjects from three separate hospitals referred for a whole-body FDG PET/CT examinations (between September 2018 and April 2019) were included in this study. A detailed breakdown of subject ages, body mass index, and clinical indications is provided in Table 1.
Recruitment	Participants were asked to be included during routine PET imaging and were included in this study if they provided informed consent)
Ethics oversight	IRB at: University of Southern California and Oregon Health Sciences University. REB et University of Toronto.

Note that full information on the approval of the study protocol must also be provided in the manuscript.
